# Use of ordinal information by fish

**DOI:** 10.1038/srep15497

**Published:** 2015-10-26

**Authors:** Maria Elena Miletto Petrazzini, Tyrone Lucon-Xiccato, Christian Agrillo, Angelo Bisazza

**Affiliations:** 1 Dipartimento di Psicologia Generale, Università di Padova, Italy; 2 Centro di Neuroscienze Cognitive, Università di Padova, Italy

## Abstract

Mammals and birds can process ordinal numerical information which can be used, for instance, for recognising an object on the basis of its position in a sequence of similar objects. Recent studies have shown that teleost fish possess numerical abilities comparable to those of other vertebrates, but it is unknown if they can also learn ordinal numerical relations. Guppies (*Poecilia reticulata*) learned to recognise the 3^rd^ feeder in a row of 8 identical ones even when inter-feeder distance and feeder positions were varied among trials to prevent the use of any spatial information. To assess whether guppies spontaneously use ordinal or spatial information when both are simultaneously available, fish were then trained with constant feeder positions and inter-feeder distance. In probe trials where these two sources of information were contrasted, the subjects selected the correct ordinal position significantly more often than the original spatial position, indicating that the former was preferentially encoded during training. Finally, a comparison between subjects trained on the 3^rd^ and the 5^th^ position revealed that guppies can also learn the latter discrimination, but the larger error rate observed in this case suggests that 5 is close to the upper limit of discrimination in guppies.

Numerical ability has rapidly become one of the most investigated topics in cognitive ethology. In the last decade, rudimentary numerical competence has been described in a large number of vertebrate species as well as in some invertebrates^1^. The capacity to discriminate between quantities can enhance survival in several ecological contexts. Choosing the patch containing the larger number of food items allows an individual to increase foraging efficiency^2^ and joining the larger social group can reduce the risks of being preyed upon by predators^3^. Numerical abilities can also be useful for deciding whether to retreat or attack a group of rivals^4^ and are very important for resource allocation during mating and reproduction^5^. The numerical skill involved in the above mentioned examples is commonly called ‘relative numerosity judgment^6^,^7^ and consists in the capacity to discriminate which groups of objects are less and more numerous. Such a capacity has been described in species as diverse as chimpanzees, *Pan troglodytes*^8^, rhesus monkeys, *Macaca mulatta*^9^, dogs, *Canis lupus familiaris*^10^, horses, *Equus caballus*^11^, parrots, *Psittacus erithacus*^12^, chicks, *Gallus gallus*^13^, and guppies, *Poecilia reticulata*^14^.

Another important numerical skill is the capacity to use ordinal information, namely the ability to locate an object on the basis of its position in a sequence (e.g., ‘four’ is larger than ‘three’ but smaller than ‘five’). Capuchin monkeys, *Cebus apella*, involved in computer tasks are able to order numerical values from one to four in ascending order and then generalise the ordinal rule to novel numerosities in the 5–9 range^15^. Likewise, rhesus monkeys can apply an ordinal numerical rule (ordering from 1 through 9) to even larger new values (10, 15, 20, and 30)^16^.

Rats, *Rattus norvegicus*, proved able to use ordinality when required to identify a target based on its numerical position^17^,^18^. In particular, subjects were trained to enter one box, of a defined ordinal number, among an array of identical boxes. Rats selected the correct target even when it occupied the 11^th^ or the 12^th^ position in a sequence of 18. Control tests ruled out the possibility that this ability was based on non-numerical cues (e.g., the overall distance to the target).

Similarly, domestic chicks were successfully trained to peck at either the 3^rd^, 4^th^, or 6^th^ position in a series of 10 locations, even when researchers controlled for non-numerical information such as the absolute distance necessary to reach the reinforced location^19^. Among birds, pigeons, *Columba livia*^20^,^21^, and parrots^22^ have also been proven capable of serial ordering.

Rudiments of ordinal abilities have been evidenced in social insects as well. Dacke and Srinivasan^23^ trained bees, *Apis mellifera*, to receive a food reward after having passed a specific number of landmarks in the experimental apparatus. Bees were able to enumerate the landmarks up to four units, including when inter-landmark distance was varied to avoid the possibility of bees locating the correct target on the basis of the absolute distance from the starting point.

Recently, there has been considerable interest in the numerical abilities of teleost fish^24^. Spontaneous choice tests and training procedures have shown that different fish species display the ability to select between larger and smaller groups and that their capacities can often compare with those of mammals and birds. Guppies, for example, can discriminate 3 from 4 social companions^25^ and can be easily trained to discriminate 4 from 5 inanimate objects^14^. As for other vertebrates, the capacity to make relative numerosity judgments permits fish to join the larger group of social companions to reduce the probability to be spotted by predators^26^, as well as it permits to select the larger group of potential prey^27^ or sexual mates^28^. However, research on fish has been entirely confined to the study of relative numerosity judgments. To date, no study has investigated whether fish can use ordinal information, even though there is no reason to believe that selective pressures in favour of the ability to process ordinal information should have acted only on mammals, birds and social insects. For instance, ordinal abilities might be adaptive for fish to solve other types of tasks, such as learning where to find a food patch in the environment (e.g., after three rocks) or where to hide from predators (e.g., after the third plant).

To address this issue, we performed four experiments. In particular we aimed to study if guppies can learn to recognise a particular feeder in a series of identical ones using exclusively its ordinal position in the sequence. In the first two experiments, guppies were trained to select the 3^rd^ feeder in a row of 8 alternative feeders, both when feeders were displaced one after the other in the direction of their route and when feeders were placed perpendicularly in front of them. To avoid the use of non-numerical spatial cues, the inter-feeder distance was experimentally manipulated. We also assessed the potential influence of olfactory cues in solving the task. In a further experiment, guppies were initially trained with both ordinal and spatial information simultaneously available, and then the two types of information were contrasted to assess which strategy they spontaneously used during the learning process. Lastly, we compared the performance of guppies trained to select the 3^rd^ or the 5^th^ feeder in a row of 12, in order to verify whether guppies’ accuracy using ordinal information decreases when learning a higher ordinal position.

## Results

### Guppies can recognize an object in a series based on its ordinal position

**Figure 1:**
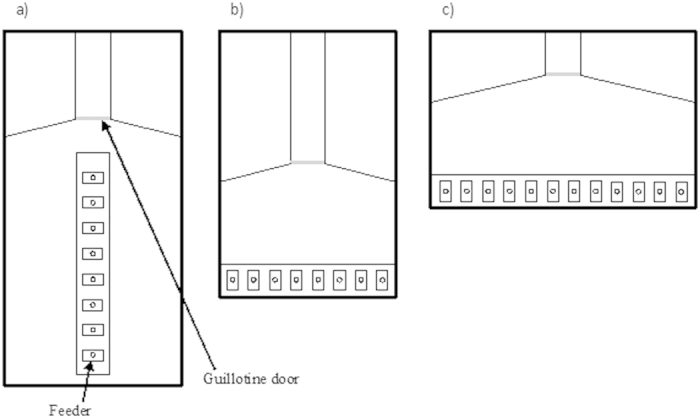
Schematic representation of the experimental apparatuses. Each trial started with the subject in the start box and the guillotine door closed. The orientation of the feeders and their number varied according to the experiment. Eight feeders were perpendicularly aligned in front of the start box but only the 3^rd^ feeder was reinforced with food (**a**). Eight identical feeders were orthogonally placed with respect to the starting point of the fish and only the 3^rd^ feeder form the left was reinforced with food (**b**). Twelve feeders were orthogonally placed with respect to the starting point of the fish and the 3^rd^ or the 5^th^ position was reinforced with food (**c**).

**Figure 2:**
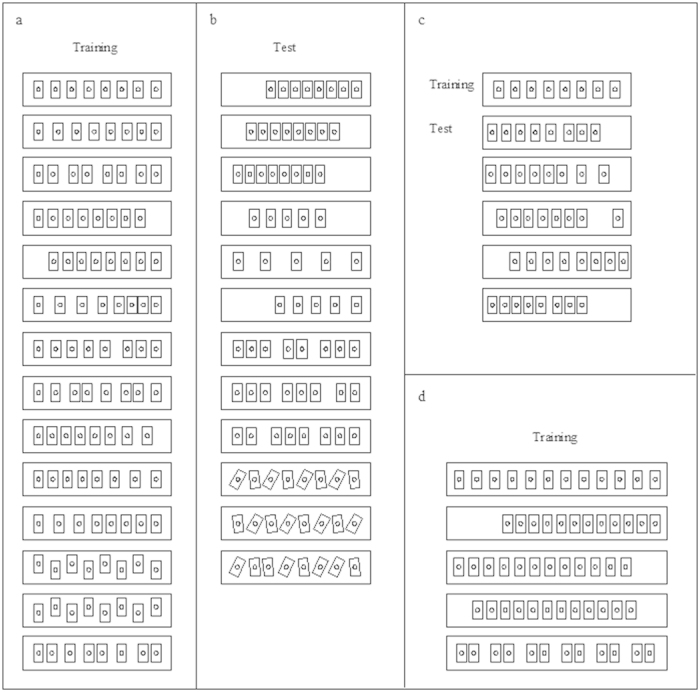
Schematic representation of the spatial arrangements used to vary the position of the feeders in the experiments. The arrangements were randomly presented according to the experimental conditions. Fourteen different arrangements were used in the training phase both when the sequence of feeder was presented in a line and when it was placed perpendicularly in front of the starting point of the subjects; inter-feeder distance and feeder positions were varied among trials to prevent the use of any spatial information (**a**). Twelve new spatial arrangements were used after the training phase to control for pattern recognition and olfactory cues (**b**). In order to verify which information guppies encode during the training, inter-feeder distance and feeder positions were held constant in the training phase so that both spatial and ordinal information were available. In the test phase new arrangements in which these two information were put in contrast were used to verify which information guppies had previously encoded (**c**). Five spatial arrangements with twelve feeders were used to compare performance of fish trained on the 3^rd^ and on the 5^th^ position (**d**).

We initially used a procedure similar to those previously adopted for studying rats, chicks and honeybees^17^,^19^,^23^. In each trial, subjects sequentially encountered 8 identical feeders placed in a line (Fig. 1a). All feeders could be opened but only the 3^rd^ feeder contained food. In order to prevent the use of non-numerical information, the relative position of the feeders changed in each trial by varying the distance between the feeders according to a pre-set scheme composed of 14 arrangements (Fig. 2a).

Three guppies (two males and one female) learned to choose the 3^rd^ feeder above chance level within the 60 trials of the Experiment 1 (chi-squared test: all p values < 0.001). The remaining female did not reach a significant performance after 6 days of training (χ^2^(1) = 0.952, p = 0.329). As it was not clear if this subject could potentially learn the task, we administrated 3 more days of training, after which the subject reached a performance significantly higher than chance (χ^2^(1) = 6.102, p = 0.014).

**Figure 3:**
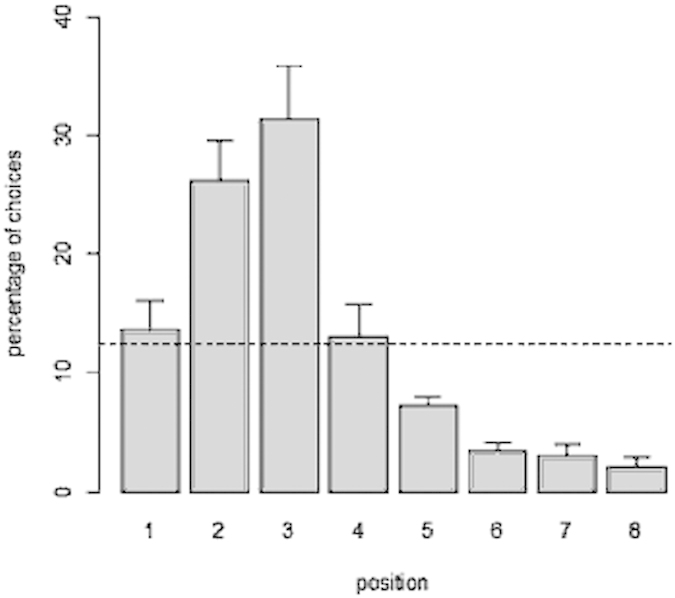
Percentage of choices for each position when guppies encountered feeders one after the other in their direction of swimming. Overall, the subjects successfully identified the 3^rd^ feeder. The dotted line represents chance level (y = 12.5%). Bars represent the standard errors.

Collectively, in the training phase the subjects chose the 3^rd^ feeder in 30.42 ± 9.47% of the trials, a preference that was significantly greater than chance (one sample t-test: t(3) = 3.786, p = 0.032) (Fig. 3). The 2^nd^ position was also selected above chance level (25.83 ± 7.01%; t(3) = 3.807, p = 0.032). No other position was significantly above chance (all p values > 0.05). In this experiment guppies did not choose the 3^rd^ feeder more often than the two adjacent ones (paired sample t-test: 3^rd^ vs. 2^nd^: t(3) = 0.577, p = 0.604; 3^rd^ vs. 4^th^: t(3) = 2.674, p = 0.075).

### Numerical precision increases when guppies can glimpse the entire series

The large imprecision shown by guppies could obviously be due to a failure to discriminate the 2^nd^ and the 3^rd^ position; however, their poor performance could also be due to the direction in which the series of objects was positioned. To distinguish between these two possibilities, in Experiment 2 we repeated the procedure with new guppies presenting 8 feeders in a row placed perpendicularly in front of the subjects (Figs. 1b and 2a).

**Figure 4:**
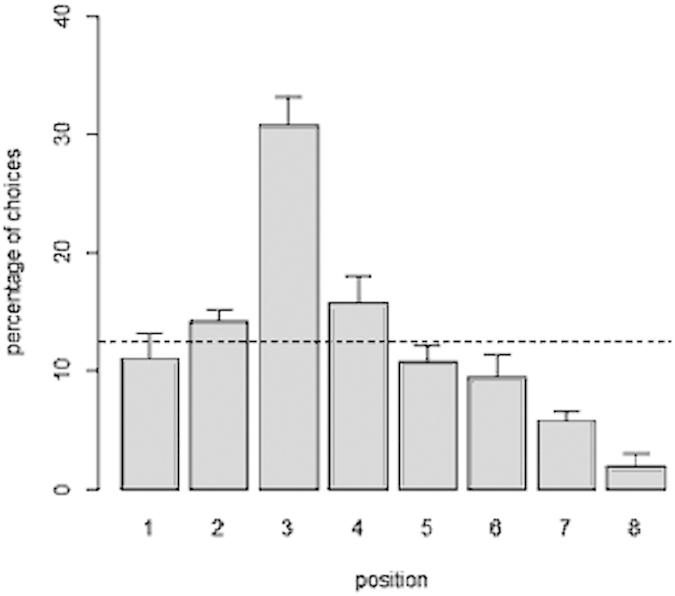
Percentage of choices for each position when the sequence of 8 feeders was placed perpendicularly in front of the starting point of the subjects. The reinforced position was the 3^rd^ from the left in the sequence. All subjects located the correct target over training. The dotted line represents chance level (y = 12.5%). Bars represent the standard errors.

All the subjects chose the 3^rd^ feeder above chance level (all p values < 0.05). Collectively, they significantly chose the 3^rd^ one (30.83 ± 5.65%; t(5) = 7.945, p = 0.001). No other position was selected above chance level (all p values > 0.05; Fig. 4). The 3^rd^ position was selected significantly more often than the two adjacent ones (3^rd^ vs. 2^nd^: t(5) = 5.533, p = 0.003; 3^rd^ vs. 4^th^: t(5) = 4.137, p = 0.009).

### Control for pattern recognition

Hypothetically subjects could have solved the latter task through pattern recognition. To rule out this possibility a test phase was performed after training where 12 totally new arrangements were presented (Fig. 2b). All the subjects chose the 3^rd^ feeder above chance level (all p values < 0.001). Collectively in the test phase, the subjects chose the 3^rd^ feeder in 39.17 ± 4.38% of the trials, a proportion significantly higher that chance (t(5) = 13.871, p < 0.001).

### Control for olfactory cues

The previous experiments could not rule out the possibility that guppies were using olfaction^29^,^30^ to locate the correct position. We then trained four new guppies to select the 3^rd^ feeder in a sequence of 8 identical ones placed in front of them (Fig. 1b) for three days followed by eight days during which we alternated reinforced trials (Fig. 2a) to probe trials (Fig. 2b) in which no food reinforcement was present.

In the test phase, all four subjects chose the 3^rd^ position above chance level (all p values < 0.001) and collectively the proportion of correct responses was significantly different from chance level (30.99 ± 7.72%; t(3) = 4.790, p = 0.017). For all four subjects, the choice of the correct feeder was significant even considering only probe trials without food (all p values < 0.05). Collectively, in these trials the subjects chose the 3^rd^ feeder in 32.81 ± 10.36%, a preference that was significantly greater than chance (t(3) = 3.920, p = 0.030). No difference was found between trials with food reward and probe trials (t(3) = 0.491, p = 0.657).

### Guppies encode the ordinal position even if other information are available

In previous experiments, we experimentally prevent fish from using non-numerical strategies (e.g., using the distance from the starting point) and thus we obliged them to use numerical information. What type of information would they use if non-numerical cues were also made available during training?

To address this issue, in Experiment 3 we trained four new guppies (Fig. 1b) with both ordinal and non-ordinal cues available by keeping feeders at the same distance and in the same position through the entire training. Then, in the test phase, we put in contrast ordinal information and spatial position of the reinforced feeder (Fig. 2c).

During the training, all four subjects chose the 3^rd^ feeder above chance level (all p values < 0.001). Collectively, they chose the 3^rd^ feeder in 41.67 ± 12.10% of the trials, a proportion that was significantly greater than expected by chance (t(3) = 4.823, p = 0.017).

In the test phase, we reinforced both the 3^rd^ feeder and the feeder in the spatial position occupied by the 3^rd^ feeder during training and all subjects chose the former above chance level (all p values < 0.001). Overall, all the subjects chose the 3^rd^ feeder in 37.50 ± 2.89% of the trials, a proportion that was significantly greater than the one expected by chance (t(3) = 17.321, p < 0.001). However, three subjects also chose above chance level the feeder in the spatial position occupied by the 3^rd^ feeder during training (all p values < 0.05), whereas one subject did not (χ^2^(1) = 0.914, p = 0.339). Overall, the subjects chose this position in the 24.38 ± 5.15% of the trials, a proportion that was significantly greater than that expected by chance (t(3) = 4.608, p = 0.019).

A direct comparison of the choice of the feeder in the correct ordinal position and the feeder in the spatially correct position showed that the subjects chose the ordinal position significantly more often (t(3) = 4.735, p = 0.018).

### Guppies decrease their precision as the ordinal position increases

A common finding in numerical cognition studies is that estimation become less precise as the numerical quantity to estimate increases^31^,^32^. We tested this hypothesis in Experiment 4 by comparing four fish trained on the 3^rd^ and four fish trained on the 5^th^ position in a row of twelve (Figs. 1c and 2d).

**Figure 5:**
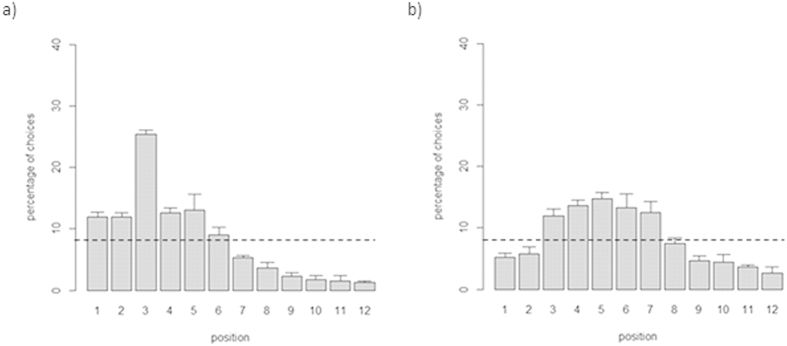
Percentage of choices for each position between subjects trained on the 3^rd^ (a) and on the 5^th^ (b) position in a sequence of twelve feeders. Overall the subjects learned to select the correct feeder, but made more errors in selecting the 5^th^ position. The dotted line represents chance level (y = 8.33%). Bars represent the standard errors.

Analysis of individual performance indicated that all fish trained on the 3^rd^ and all fish trained on the 5^th^ position performed the task above chance levels (all p values < 0.05). Collectively, the subjects trained on the 3^rd^ position chose the reinforced feeder in 25.38 ± 1.40% of the trials, whereas the subjects trained on the 5^th^ position chose the reinforced feeder in 14.81 ± 1.92% of the trials. Both proportions were significantly greater than expected by chance (3^rd^ position: t(3) = 28.283, p < 0.001; 5^th^ position: t(3) = 6.734, p = 0.007) but subjects trained on the 3^rd^ position had a better performance than those trained on the 5^th^ position (independent t-test: t(6) = 8.883, p < 0.001). Subjects trained on the 3^rd^ feeder chose the reinforced target significantly more often than the two adjacent positions (2^nd^ feeder: 11.92 ± 1.47%; t(3) = 16.813, p < 0.001; 4^th^ feeder: 12.69 ± 1.47%; t(3) = 12.473, p = 0.001) (Fig. 5a). In contrast, the subjects trained on the 5^th^ feeder did not choose the reinforced feeder significantly more often than the two adjacent ones (4^th^ feeder: 13.65 ± 1.71%; t(3) = 0.812, p = 0.476; 6^th^ feeder (13.08 ± 4.66%; t(3) = 0.827, p = 0.469) (Fig. 5b).

### Sex differences

In all our experiments we used both males and females. This permits an exploratory analysis of sex differences in ordinal abilities. Because of the small sample size used in each experiment, we analysed the pooled data of the training phase of all the experiments (16 males and 10 females) using the standardised score of the proportion of correct responses, as the experiments differ with respect to the number of trials. The average standardised score did not significantly differ between sexes (females: 0.14 ± 1.37 (mean ± s.d.), males: 0.21 ± 0.81; t(24) = 0.816, p = 0.423).

## Discussion

Teleost fish have been found to be very efficient in the discrimination of numerical quantities, showing abilities comparable to those of many mammals and birds^14^,^33^. However it is not known if these similarities extend to other numerical abilities. Rhesus macaques^16^, rats^18^, chicks^19^, pigeons^20^ and bees^23^ are able to use ordinal information. Our results show that guppies too can use ordinal information to identify a specific position in a series of otherwise identical objects.

In this study, guppies learned to identify the 3^rd^ feeder in a row of 8 identical alternative feeders in a relatively small number of trials, both when they encountered feeders one after the other in their direction of swimming and when feeders were perpendicularly aligned with respect to the starting point of the fish. However, the performance was much more accurate in the latter condition. With sequentially encountered feeders while swimming along the sequence of feeders, a subject progressively loose the capacity to glance the entire series of objects and it is possible that the need to keep in mind the number of non-visible objects made the task more difficult for guppies. The higher number of errors on the second position also suggests that guppies might be unable to completely inhibit the tendency to dislodge the disc when they encounter the feeder preceding the correct one. Control tests using a larger inter-feeder distance presented in their direction of swimming are necessary to test this hypothesis.

The position of an object in a series can be learned using a non-numerical strategy. For instance an animal can learn to stop at a given distance from the first object of the row or swimming from the starting point in a precise direction. Fish are also able to use specific landmarks and the geometry of the environment to precisely locate a point in space^34^,^35^. To prevent this possibility, during the training phase the absolute position of the rewarded feeder was varied across trials. In the second experiment guppies proved able to choose the 3^rd^ position more often than the adjacent ones, indicating that they could rely on solely the ordinal position to identify the location of the reinforced feeder. However, fish are known to possess sophisticated learning skills and can discriminate complex patterns^36^,^37^. Blind Mexican cavefish (*Astyanax fasciatus*) for example can precisely learn the relative position of four landmarks placed in their environment and they change their exploratory behavior when the position of two adjacent landmarks is switched^38^. Our subjects could have solved the task by learning all the spatial arrangements or, at least, some of them. To test for this hypothesis, we did a control test presenting 12 novel spatial arrangements. Even in this case guppies selected the 3^rd^ feeder thus excluding that pattern recognition played a key role in solving the task.

Several fish species, especially those active at night or living in turbid water, are known to use the olfactory system to search for food^29^,^30^. This has never been demonstrated in guppies, but these fish have been shown to respond to chemical cues from conspecifics and predators^39^. One may argue that olfactory cues may have guided the subjects’ decisions during our experiments. However, the finding that subjects trained to find food on the 3^rd^ position still chose the correct feeder in the absence of a food reward excludes this possibility. This also aligns with previous studies on guppies using similar feeders, in which no influence of olfactory cues was found^14^,^40^. That said, it is worth noting that we made every effort to avoid the possibility of fish using olfaction to locate the food in our experiments, but we cannot exclude that guppies in nature might integrate olfactory and visual information to locate a hidden food source.

Some authors have suggested that animals are not naturally attuned to number, as numerical information would be less salient in natural contexts than physical attributes; as a consequence, animals would be expected to use number only as a last-resort strategy, when no other cues are available to discriminate between quantities^41^,^42^. This hypothesis is supported by evidence showing that mosquitofish, *Gambusia holbrooki*, could easily select the larger group of social companions, but their performance dropped to chance level when the cumulative surface area between shoals was controlled for^43^. However, when prevented from using non numerical attributes, mosquitofish proved able to select the larger shoal relying exclusively on numerical information, in agreement with the last-resort hypothesis^26^. This does not occur in all cases. Agrillo *et al.* (2011)^44^ found that mosquitofish trained to discriminate bi-dimensional figures showed no difference in learning rates when allowed to use continuous quantities only or numbers only. However, they learned more quickly when these two kinds of information were simultaneously available, in accordance with previous literature showing how redundancy of information facilitates learning both in humans and in non-human animals^45^,^46^. The same problem exists for discrimination of ordinal position. To address this issue, we repeated the procedure used in the second experiment but maintained constant spacing among the feeders and their absolute spatial position during the whole training period, so that the guppies were free to use ordinal or spatial information (or both) to learn the task. In the test phase, when the ordinal position was contrasted with the spatial position, fish chose the 3^rd^ feeder (the correct ordinal position) significantly more often than the feeder occupying the spatial position held by the feeder reinforced during training. However, 3 out of the 4 subjects also chose the spatially correct position above chance, indicating that, to some extent, guppies were also encoding spatial position during the learning phase. Our data therefore suggest that, for a guppy in an ordinal-position task, numerical information may be easier to be processed than other types of information (i.e., spatial positioning or the spatial relationships among different objects); thus, the argument that fish use number only as a last-resort is not supported in the context of ordinal abilities. In addition, the fact that spatial cues seem to be more salient than numerical information in relative numerosity judgments of fish^43^,^47^,^48^ while the opposite result was found in this ordinal task reinforces the idea – previously advanced in the literature – that the relative salience of numerical information over spatial cues is context-dependent^49^.

The tendency to rely more on ordinal information than on other cues might have a potential adaptive value in nature for a species living in shallow waters, where the relative position of stable elements of the landscape could be used as cues for orienting in space as observed in other fish, including species of the same family^50^. For example, guppies could learn that there is a food patch after three consecutive rocks or use a specific position in a row of similar trees on the bank for orientation while escaping from predators. The shape and the details of these objects are more likely to change over seasons than their relative position.

From the results of this experiment it is also clear that, although guppies privileged ordinal information, at least some individuals were in fact making use of multiple types of information (spatial and ordinal) to solve the task. In nature, numerical and continuous information are often simultaneously available: more pieces of food have a greater cumulative surface, larger social groups occupy a larger volume, and more calls require more time to be produced^43^,^51^,^52^. Not surprisingly, guppies and other vertebrates have evolved mechanisms that use redundant information to estimate quantities^44^,^53^.

Several authors agree with the existence of an approximate number system (ANS) able to support the representations of numerosities over the whole numerical range. Numerical discriminations produced through this mechanism are subject to Weber’s law: errors generally increase in proportion with increasing numerosity, and accuracy is determined by the magnitude of the set to be enumerated^31^,^32^. The possibility exists that performance in ordinal tasks is similarly affected by the number of items, such that the ability to locate the correct target decreases as the ordinal position increases. For instance, bees can identify up to 4 positions in a series of different alternatives but beyond the 4^th^ position they are no longer able to identify the correct landmark^23^. Conversely, studies on rats and chicks did not find a similar decrease in discriminability as a function of the ordinal position^18^,^19^. We addressed this issue by comparing the training on the 3^rd^ and 5^th^ positions in a row of 12 identical feeders. Fish trained on the 5^th^ feeder still located the correct target but were less accurate than fish trained on the 3^rd^ feeder, as they made more attempts at adjacent feeders compared to the other group. This finding might suggest that 5 units could be the upper limit of guppies’ ability to use ordinal information, the same numerical threshold recently reported in cardinal tasks^14^. Although future studies in which guppies are trained to select ordinal positions higher than 5 are required to confirm this hypothesis, it seems clear that the performance of guppies does not attain that of other vertebrates (e.g., rats are able to learn up to the 12^th^ position). The most obvious explanation for this difference resides in the enormous brain development of mammals and birds, which are both superior to fish in many other functions^54^. However, we cannot exclude that different findings may be ascribed to the different methodologies used across the studies (e.g., learning criteria, number of trials, etc.). It has been shown that numerical acuity within the same species can greatly vary as a function of the methodology adopted^55^. Another possibility is that inter-species differences reflect distinct selective pressures that acted on each species due to adaptation to their respective ecological niches. For instance, rats are known to dig extensive burrow systems consisting of food storage chambers and feeding sites^56^. It is possible that the foraging behaviour of this species has favoured the development of sophisticated abilities to locate food in different chambers on the basis of their position in the burrow network.

In summary, our experiments provide the first evidence that guppies can use ordinal numerical information, even when alternative non-numerical cues are available. Our findings, together with results in other distantly related species, point to the existence of numerical ordinal competencies widespread among non-human animals. Future studies are now needed to shed more light on ordinal abilities of guppies. For instance, a recent study suggests that birds exhibit a spontaneous leftward bias when required to locate an object by using ordinal information, indirectly evoking the idea of a mental number line similar to that described in humans^57^. In our study we did not attempt to assess whether also fish preferentially enumerate from left to right or vice versa, a hypothesis that should be tested in the near future. Also, further research is welcome to assess whether this ability is unique to guppies or instead is shared by other fish species living in different ecological contexts—for instance, in pelagic fish that do not normally experience stable sequences of objects in their environment.

## Methods

### Subjects

We used adult males and females of an ornamental guppy stock (snakeskin cobra green) maintained at the Department of General Psychology in 150-liter glass aquaria. Each aquarium was provided with abundant natural and artificial plants, water filters and 15 w fluorescent lamps ensuring light from 8:00 to 20:00. Fish were fed with commercial food flakes and *Artemia salina* nauplii, three times a day. The experimental subjects were randomly selected from the maintenance tanks. Each subject participated in only one experiment. Experimental tanks were provided of natural plants, bottom gravel and social companions to minimize differences from maintenance tanks. At the end of the experiment, subjects were released in maintenance tanks identical to the ones previously described, and kept only for breeding purpose. Experiments comply with the law of the country (Italy) in which they were performed (*Decreto legislativo 4 marzo 2014*, n. 26). The experimental procedure has been approved by the Animal Ethical committee of the *Università di Padova* (permit N° 35/2013) and the *Organismo Preposto per il Benessere degli Animali*, *Università di Padova* (O.P.B.A., permit prot. N° 116039).

### Apparatus

Experiments were conducted in glass tanks filled with natural gravel and 30 cm of water. The size of the tanks and the apparatus slightly varied for each experiment (40 × 80 × 35 cm, Fig. 1a; 40 × 60 × 35 cm, Figs. 1b and 1c). Each tank was divided into an experimental compartment, in which the trials took place, and a start box made of green plastic material. In the experimental compartment, opposite the start box, a light yellow panel was placed horizontally on the gravel substratum. The panel accommodated plastic feeders used for the experiments. Each feeder (2.5 × 4.5 cm, height 1.5 cm) had a small central hole (Ø 1 cm, depth 0.5 cm). In the far end of the start box, abundant natural plants created a natural-like environment. A transparent guillotine door, controlled remotely by the experimenter, regulated the movements of the subject between the experimental compartment and the start box. During the collective habituation to the procedure, we used a habituation tank identical to the experimental one, but a green plastic panel (20 × 15 cm) with 83 holes (Ø 1 cm, depth 0.5 cm) was placed on the gravel substratum.

### Description of an experimental trial

We adopted a training procedure previously used to study learning abilities in guppies^14^,^40^. Each trial started with the subject in the start box, with the transparent guillotine door closed. A green plastic panel was placed in front of the transparent door to prevent sight during the set-up of the trial.

The plastic feeders in the experimental compartment were then placed in a row and spaced out according to the schedule of each trial. The holes were covered by yellow plastic discs (diameter 1.2 cm, height 0.2 cm). A small quantity of commercial food flakes, crumbled, was concealed in the hole of the feeder in the position to be reinforced. Water scented with food was added to the experimental tank prior to each series of trials, both to motivate the subject to search for food and to mask olfactory cues to the location of the reward. The green plastic panel was then removed and the subject was allowed to observe the experimental compartment for 10 seconds prior its release. The choice of the subject was scored as the first disc dislodged. One minute was granted to the subject to consume the food after finding it. We used a partial correction procedure: if the subject made a wrong choice, it was allowed to dislodge up to two more discs in each trial, after which it was gently conducted to the start box using a transparent plastic panel. If the subject did not find the food reward within 10 minutes, the trial was ended. In each successive trial, the feeder containing the food was replaced with a clean one and the relative position of the feeders was randomly changed.

### Experimental phases

The procedure followed 5 sequential phases. Phases 1–3 were aimed to habituate the subjects to the experiment and to train them to dislodge the discs. Learning performance of the subjects was evaluated in phases 4 and 5.

Phase 1—Collective habituation to the apparatus: Two subjects were moved from the maintenance aquarium to a tank similar to the experimental apparatus for 3 days of habituation.

Phase 2—Collective habituation to the procedure: The subjects were familiarised with the experimental procedure for 2 days. Two times per day, they were conducted to the start box as in the trial procedure. The experimenter supplied a small quantity of food in 5 holes of the plate, randomly chosen, and then allowed the subjects to enter the experimental compartment to feed. At the end of the second day, each subject was individually moved to the experimental apparatus, where two adult and five sub-adult conspecifics were present to avoid social isolation. During the trials, these social companions were moved in another tank and received their food ration. After 1 day of habituation to the experimental apparatus, the subject started the experiment.

Phase 3—Training to dislodge the discs: During the first day of the experiment, the subject performed 8 trials (4 in the morning and 4 in the afternoon) in which food was delivered in the hole of the reinforced feeder but no disc was present. On day 2, the subjects performed 8 trials in which the discs were present on all feeders, but they only partially covered the holes. Only the hole of the reinforced feeder contained food. The proportion of the hole covered by each disc was increased across trials, from 25% in trials 1–3, to 50% in trials 4–6, to 75% in trials 7–8. On the third day, the subjects performed 10 trials (5 in the morning and 5 in the afternoon). In trials 1, 2, 6, and 7 the discs covered 75% of each hole. In trials 3–5 and 8–10 only the disc of the reinforced feeder was present and it completely covered the hole. Due to the greater difficulty of the task, in the last experiment this exact sequence was repeated on the following day. Phase 3 was not considered in data analysis. To ensure high motivation, from phase 3 onward, subjects were not fed except during the experiment. As we adopted a partial correction procedure, they each received a daily ration roughly equivalent to the food a fish typically receives in the housing conditions. Eight subjects were excluded from the experiments in this phase: 3 refused to leave the start box to participate in the trials, and the other 5 never learned to dislodge the discs.

Phase 4—Training: Each day, the subjects performed 5 trials in the morning and 5 trials in the afternoon. In each trial, the hole of each feeder was completely covered by a disc. In the first 3 days of this phase, an additional cued trial (not considered in the analyses) was performed in the morning, 30 minutes before the experiment. In this trial, only the feeder in the reinforced position was covered by the disc and contained food. In a pilot experiment this procedure was found to be necessary; because there were 7 or more empty feeders in each trial and just one feeder containing food, before the task was learned, fish were likely to experience a sequence of failures and hence lose interest in the experiment.

Phase 5—Test phase: An additional test phase was performed in Experiments 2 and 3. A detailed report of this phase is given in the description of each experiment.

### Experiment 1. Ordinal position in a sequentially encountered series of feeders

Two male and 2 female guppies participated in the experiment. In each trial, the subjects encountered sequentially 8 identical feeders placed in a line (Fig. 1a). All feeders could be opened but only the 3^rd^ feeder contained food.

The training (phase 4) lasted 6 days. In phases 3 and 4, 8 feeders were presented in each trial but only the 3^rd^ feeder was reinforced with food. In order to prevent the use of non-numerical information, the relative position of the feeders changed in each trial according to a pre-set scheme composed of 14 arrangements (Fig. 2a). In particular, we systematically varied the distance between the reinforced feeder and the start box and the spacing between feeders.

### Experiment 2. Ordinal position when the series of feeders was placed in front of the subject

Four male and 2 female guppies participated in the experiment. The experimental procedure was the same as Experiment 1 except that the row of feeders was rotated by 90° and placed perpendicularly in front of the starting point of the subjects (Fig. 1b). The reinforced feeder was the 3^rd^ from the left. As in the previous experiment, to avoid the use of non-numerical cues, the position of the reinforced feeder was changed in each trial according to a pre-set scheme composed of 14 arrangements (Fig. 2a).

### Control for pattern recognition

The training phase of this experiment was followed by a test phase lasting 4 more days. The procedure was the same but we presented 12 new arrangements of the feeders (Fig. 2b) to control for the possibility that the subjects had solved the task by using pattern recognition (i.e., by learning the position of the food in the 14 arrangements presented during training). Three of the 12 arrangements were made using only 5 feeders, allowing an increase in the variance of the spatial position of the reinforced feeder. One male ceased to participate after the second day of the control phase. For this fish statistics were calculated on the 20 trials it performed.

### Control for olfactory cues

To assess whether guppies could use olfactory cues to find the correct feeder, four new fish (3 male and 1 female) were trained as in previous experiment and, after the training phase, they performed a series of extinction trials in which no food reinforcement was present. The training (phase 4) lasted 3 days. The test phase (phase 5) lasted 8 days, during which the subjects performed 12 trials per day—8 normally reinforced on the 3^rd^ position (Fig. 2a) and 4 in extinction (Fig. 2b). The extinction trials were never conducted at the beginning or the end of the sequence, and were never consecutive.

### Experiment 3. Encoding of ordinal position when other information are available

Two male and 2 female guppies were trained to the 3^rd^ position for 6 days as previously (Fig. 1b). However in this experiment during the training, the feeders were evenly spaced (distance 4.5 cm) and their position was held constant (Fig. 2c) so that both spatial and ordinal information were available to solve the task. To verify which information guppies had encoded in the training phase, in the test phase, we tested guppies with new arrangements of the feeders (Fig. 2c) in which these two information were put in contrast. In particular, the 3^rd^ feeder was always in a spatial position different from that occupied during training and its spatial position was occupied by the 1^st^, 2^nd^ or 4^th^ feeder. In each trial, food reward was placed both in the 3^rd^ feeder and in the feeder that occupied the position previously occupied by the 3^rd^ feeder.

### Experiment 4. Comparison of fish trained on 3^rd^ and 5^th^ position

We trained eight guppies. The training phase in this experiment lasted 13 days and we presented 12 feeders in each trial (Fig. 1c). Half of the subjects (3 males and 1 female) were trained on the 3^rd^ feeder, whereas the other half (2 males and 2 females) were trained on the 5^th^ feeder. The position of the feeders varied in each trial according to a pre-set scheme, in order to prevent the use of non-numerical information (Fig. 2d).

### Statistical analysis

In all the experiments, we performed both individual and group analyses. Individual performance was firstly analysed through chi-square tests on frequencies of choices. Subsequently, overall performance of the subjects was assessed using t-tests on the proportion of correct choices. Analyses were carried out using R statistical software (version 3.0.2; R Development Core Team). In the text, percentages of choices (mean ± s.d.) are given.

## Additional Information

**How to cite this article**: Miletto Petrazzini, M. E. *et al.* Use of ordinal information by fish. *Sci. Rep.* **5**, 15497; doi: 10.1038/srep15497 (2015).
